# An altered gene expression profile in tyramine-exposed intestinal cell cultures supports the genotoxicity of this biogenic amine at dietary concentrations

**DOI:** 10.1038/s41598-018-35125-9

**Published:** 2018-11-19

**Authors:** Beatriz del Rio, Begoña Redruello, Victor Ladero, Santiago Cal, Alvaro J. Obaya, Miguel A. Alvarez

**Affiliations:** 1 Dairy Research Institute (IPLA-CSIC), Paseo Rio Linares s/n, 33300 Villaviciosa, Spain; 2 Instituto Universitario de Oncología (IUOPA), Universidad de Oviedo, Av. Julián Clavería, s/n, 33006 Oviedo, Spain; 3 Dpto. de Bioquímica y Biología Molecular, Universidad de Oviedo, Av. Julián Clavería, s/n, 33006 Oviedo, Spain; 4 Dpto. de Biología Funcional, Universidad de Oviedo, Av. Julián Clavería, s/n, 33006 Oviedo, Spain

## Abstract

Tyramine, histamine and putrescine are the most commonly detected and most abundant biogenic amines (BA) in food. The consumption of food with high concentrations of these BA is discouraged by the main food safety agencies, but legal limits have only been set for histamine. The present work reports a transcriptomic investigation of the oncogenic potential of the above-mentioned BA, as assessed in the HT29 human intestinal epithelial cell line. Tyramine had a greater effect on the expression of genes involved in tumorigenesis than did histamine or putrescine. Since some of the genes that showed altered expression in tyramine-exposed cells are involved in DNA damage and repair, the effect of this BA on the expression of other genes involved in the DNA damage response was investigated. The results suggest that tyramine might be genotoxic for intestinal cells at concentrations easily found in BA-rich food. Moreover, a role in promoting intestinal cancer cannot be excluded.

## Introduction

Biogenic amines (BA) are nitrogenous organic bases produced by practically all living organisms via the enzymatic decarboxylation and deamination of the corresponding amino acids^1,2^ - histamine from histidine, tyramine from tyrosine, etc. BA can accumulate at high concentrations in certain foods, e.g., fish, fish products, fermented foods, fermented beverages, owing to the presence of microorganisms with the corresponding amino acid decarboxylase activities. Despite BA being involved in important biological processes, they can cause toxic reactions when ingested in high concentrations. The presence of BA in food therefore poses a risk to human health^2–4^. Tyramine, histamine and putrescine, the BA most commonly found in BA-rich food^5,6^, may accumulate at very high concentrations in the above-mentioned foodstuffs^2,7–10^.

The European Food Safety Authority (EFSA) considers tyramine and histamine to be the most toxic of all dietary BA, and although legal limits have only been set for histamine, it deems all of them to represent an important food safety risk^10^. It is well known, for example, that the consumption of cheese containing high amounts of tyramine can cause the so-called “cheese reaction”, the symptoms of which include hypertension, headache, and even migraine^2,3,11^. Similarly, the ingestion of food with a high concentration of histamine can cause symptoms resembling an allergic reaction, producing urticaria, gastrointestinal and respiratory problems, tachycardia and hypotension. It is often referred to as histamine or “scombroid” poisoning^2,3,12^. Putrescine is also understood to pose a threat to human health^10^. It can potentiate the toxic effects of histamine by competing with it for the enzymes involved in the latter’s detoxification in the intestinal mucosa (diamine oxidase and N-methyltransferase)^7^, and may also increase the absorption of histamine into the bloodstream^13^. Dietary putrescine has also been associated with cancer, since it can form carcinogenic nitrosamines when it reacts with nitrites present in food^2,14^. Certainly it has been linked (along with the polyamines spermine and spermidine) to the appearance of colorectal adenoma^15^.

In 2011, the EFSA Panel on Biological Hazards (BIOHAZ) highlighted our scant knowledge of the toxicity of tyramine and histamine and of their concentrations in foods, and warned of their potentiation of the effects of putrescine and cadaverine (two other dietary BA)^10^. Since then, much more has been learned about the cytotoxicity of tyramine and histamine (the most toxic dietary BA); our knowledge of putrescine and cadaverine has also been improved. We recently showed that tyramine, histamine, putrescine and cadaverine are cytotoxic towards a human intestinal epithelial cell line at concentrations commonly reached in BA-rich food^16^ (del Rio *et al*., submitted). In addition, tyramine and histamine showed a synergistic cytotoxic effect^17^ - a food safety concern given that these BA are commonly found together at high concentrations in certain BA-rich food (e.g., cheese)^5,6,9^. This *in vitro* model also allowed the modes of action of these BA to be assessed; tyramine, putrescine and cadaverine were shown to induce cellular necrosis, while histamine causes apoptosis^16^ (del Rio *et al*., submitted). The molecular basis of the cytotoxicity of tyramine and histamine needs to be further investigated, as does the potential cytotoxicity of putrescine and cadaverine.

Gene expression profiling may help us better understand the molecular response of cells exposed to BA. Using a human intestinal cell line, and a real-time quantitative PCR (qPCR) array covering 84 genes associated with different oncogenic pathways, the present work examines the possible oncogenic potential of tyramine, histamine and putrescine via the changes in the expression profile of genes involved in tumorigenesis. Tyramine had the greatest effect on gene expression. Since some of the genes showing a significantly different level of expression after tyramine exposure have been implicated in DNA damage-signalling and repair, the effect of this BA on the expression of genes involved in these processes was examined using a further real-time qPCR array.

## Results

### Effect of tyramine, histamine and putrescine on the expression of genes involved in oncogenic pathways

The RT^2^ PCR Human Cancer Pathway Finder array (which covers 84 genes implicated in oncogenesis) was used to assess whether tyramine, histamine and putrescine perturb any biological pathways involved in human cell transformation and tumour formation. HT29 cells were treated for 6 h with 14.58 mM tyramine (equivalent to 2000 mg/kg), 17.99 mM of histamine (equivalent to 2000 mg/kg) and 17.02 mM putrescine (equivalent to 1500 mg/kg) - the highest concentrations ever reported in food^10^. Untreated HT29 cell cultures were used as controls. Total RNA was isolated from both the BA-treated cells and the control cultures, and the relative expression of the genes covered by the array was determined. The full set of results obtained are shown in Supplementary Table S1.

**Figure 1:**
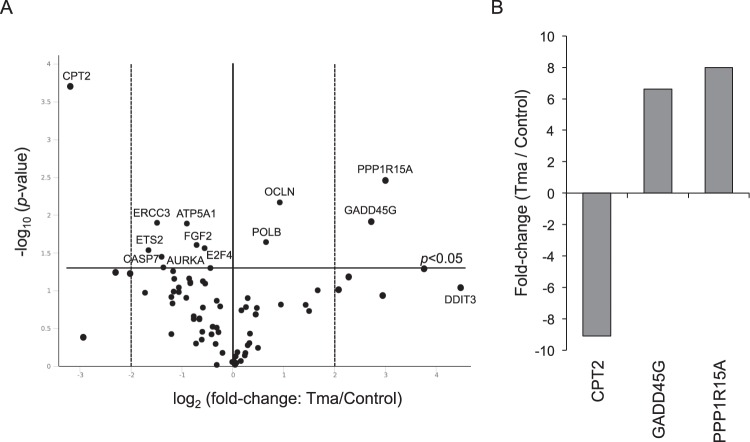
Gene expression analysis of cancer pathways in tyramine-treated HT29 cells compared to untreated cells. (**A**) Volcano plot representation of tyramine-exposed HT29 cell cultures vs. untreated control cultures. The horizontal black line represents the threshold of statistical significance (*p* = 0.05). The vertical dotted black lines represent a fold change cut-off of ≥4. (**B**) Genes showing a statistically significant fold change (≥4) are shown. See also Supplementary Table 1S. Tma: tyramine.

The tyramine-exposed HT29 cells showed 12 genes with a significantly (*p* < 0.05) altered expression (Fig. 1A). Four were upregulated - PPP1R15A (7.98-fold), GADD45G (6.63-fold), OCLN (1.90-fold) and POLB (1.58-fold) - and eight were downregulated - CPT2 (9.09-fold), ETS2 (3.15-fold), ERCC3 (2.80-fold), CASP7 (2.62-fold), AURKA (2.57-fold), ATP5A1 (1.87-fold), FGF2 (1.64-fold) and E2F4 (1.46-fold) (Fig. 1A and Supplementary Table S1). ERCC3, GADD45G, POLB and PPP1R15A are involved in DNA damage-signalling and repair, AURKA and E2F4 are involved in cell cycle regulation and progression, ETS2 in cellular senescence, FGF2 in angiogenesis, OCLN in epithelial-mesenchymal transition, CASP7 in apoptosis, and ATP5A1 and CPT2 in metabolism. Figure 1B shows those genes with an expression fold change of ≥4 (CPT2, GADD45G and PPP1R15A). The DDIT3 gene, which is also involved in DNA damage-signalling and repair, appeared to be upregulated very strongly − 22.37-fold (Supplementary Table S1) - but the change was not statistically significant (*p* = 0.0924).

Exposure to histamine significantly modified the expression of four genes: ARNT was upregulated (1.80-fold), while CPT2 (2.95-fold reduction), CFLAR (1.40-fold reduction) and E2F4 (1.36-fold reduction) were downregulated (Supplementary Table S1). ARNT is involved in hypoxia signalling, CFLAR in apoptosis, CPT2 in metabolism, and E2F4 in cell cycle regulation.

Exposure to putrescine affected just two genes, both of which were downregulated: CPT2 (2.18-fold) and PPP1R15A (1.6-fold) (Supplementary Table 1S). As stated above, PPP1R15A is involved in DNA damage-signalling and repair, while CPT2 is involved in metabolism.

Since none of the genes affected by histamine or putrescine showed an expression fold change of ≥4, all further work was focused on tyramine.

### Effect of tyramine on the expression of genes involved in DNA damage-signalling pathways

**Figure 2:**
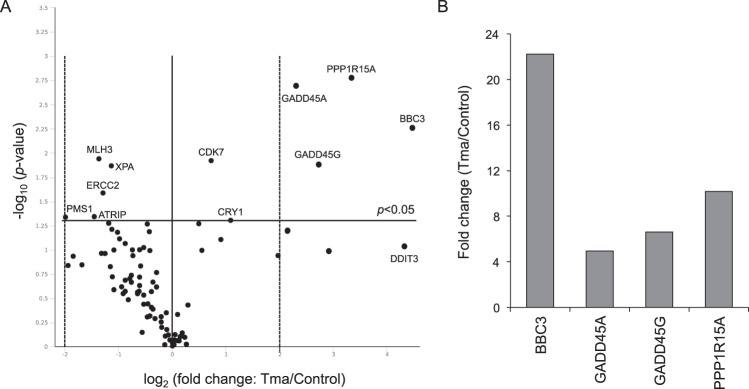
Gene expression analysis of DNA damage-signalling pathways in tyramine-exposed HT29 cells compared to untreated cells. (**A**) Volcano plot representation of tyramine-exposed HT29 cultures vs. untreated control cultures. The horizontal black line represents the threshold of statistical significance (*p* = 0.05). The vertical dotted black lines represent a fold change cut-off of ≥4. (**B**) Genes showing a statistically significant fold change (≥4) are shown. See also Supplementary Table 2S. Tma: tyramine.

As shown above, tyramine modified the expression of four genes involved in DNA damage-signalling and repair. Two of these showed an expression fold change of ≥4 (GADD45G and PPP1R15A). Work was therefore performed to determine whether tyramine modifies the expression of other genes involved in the DNA damage response. This was done using the RT^2^ PCR Human DNA Damage Signaling Pathway Array, which includes genes associated with the ATR/ATM signalling network and the DNA damage response. HT29 cells were treated as described above with 14.58 mM tyramine. Total RNA was then isolated from treated and control cultures, and the relative expression of the 84 genes covered by the latter array determined. Supplementary Table S2 shows the results obtained. Figure 2A shows a volcano plot of the differentially expressed genes. Six were upregulated - BBC3 (22.22-fold), PPP1R15A (10.19-fold), GADD45G (6.64-fold), GADD45A (4.94-fold), CRY1 (2.14-fold) and CDK7 (1.65-fold) - and five were downregulated - PMS1 (3.94-fold), ATRIP (2.73-fold), MLH3 (2.56-fold), ERCC2 (2.44-fold) and XPA (2.19-fold). XPA and ERCC2 are involved in nucleotide excision repair (NER), MLH3 and PMS1 in mismatch repair (MMR), ATRIP, CRY1, GADD45A and GADD45G in DNA repair, BBC3 and PPP1R15A in apoptosis, ATRIP, CDK7 and PPP1R15A in cell cycle regulation, and ATRIP in ATM/ATR signalling. Among these, BBC3, GADD45A, GADD45G and PPP1R15A showed an expression fold change (all upregulated) of ≥4 (Fig. 2B). DDIT3 again showed apparently strong but non-significant (*p* = 0.0927) upregulation (22.20-fold; Supplementary Table S2) compared to control cultures.

### Validation of the RT^2^ PCR array data by real-time quantitative PCR analysis (qPCR)

**Figure 3:**
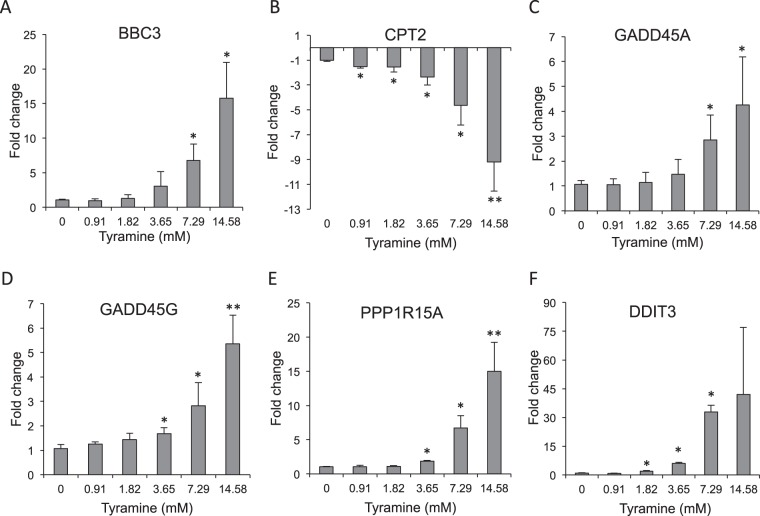
Real time qPCR gene expression analysis of differently expressed genes – (**A**) BBC3, (**B**) CPT2, (**C**) GADD45A, (**D**) GADD45G, (**E**) PPP1R15A and (**F**) DDIT3 - in tyramine-exposed HT29 cells compared to control cultures. Cell cultures were exposed to one of six tyramine concentrations (0, 0.91, 1.82, 3.65, 7.29 or 14.58 mM) for 6 h. Data were normalized against the total RNA content using GAPDH as a reference gene. The data represent the mean of two biological replicates (each in duplicate). Vertical bars represent standard deviations. *p < 0.05, **p < 0.001 compared to control cultures.

The differences in gene expression obtained using the RT^2^ PCR arrays were validated by real time qPCR (qPCR) analysis, exposing cells to increasing concentrations of tyramine (0.91 mM to 14.58 mM). Analyses were performed for the five genes showing an expression fold-change of ≥4 in the above array analysis: BBC3, CPT2, GADD45A, GADD45G and PPP1R15A. Since DDIT3 showed apparently strong (although non-significant) upregulation (22.37-fold with the Human Cancer Pathway Finder Array and 22.20-fold with the Human DNA Damage Signaling Pathway Array), it was also analyzed by qPCR. BBC3, GADD45A, GADD45G, PPP1R15A and CPT2 all showed dose-dependent expression changes, validating the array-based findings (Fig. 3). BBC3 and GADD45A showed a significant upregulation when cells were exposed to tyramine concentrations above 7.29 mM (Fig. 3A,C), while GADD45G and PPP1R15A showed significant upregulation at concentrations above 3.65 mM (Fig. 3D,E). CPT2 showed a significant downregulation even at the lowest tyramine concentration assayed (0.91 mM) (Fig. 3B). DDIT3 showed a dose-dependent upregulation (Fig. 3F), significantly so at tyramine concentrations of 1.82, 3.65 and 7.29 mM. Surprisingly, at the 14.58 mM concentration, the expression change was not significant due to great variability among the biological replicates.

## Discussion

The present work aimed to assess the possible oncogenic potential of tyramine and histamine - the most toxic dietary BA^10,16,17^ - and putrescine, which is also considered a hazard for human health^10^. Gene expression profiling was used to explore whether these BA affect the expression of genes involved in tumorigenesis and the malignant transformation of human intestinal cells. Studies were performed in confluent HT29 cell cultures with the characteristics of mature intestinal cells (forming a monolayer with tight junctions between the cells and a typical apical brush border resembling that of the gastrointestinal mucosa^18^).

The present results show that tyramine can significantly repress the expression of CPT2. This gene codes for carnitine palmitoyltransferase II, an enzyme of the inner mitochondrial membrane involved in fatty acid ß-oxidation for the production of ATP^19^. Some studies report a correlation between CPT2 repression and carcinogenesis. For instance, the repression of CPT2 in hepatocytes was reported to be associated with abnormal lipid accumulation due to the suppression of fatty acid ß-oxidation, inducing the malignant transformation of hepatocytes^20^ and hepatocarcinogenesis^21^. It might therefore be speculated that the repression of CPT2 by tyramine also leads to abnormal lipid accumulation, perhaps contributing towards the malignant transformation of intestinal epithelial cells.

Although tyramine treatment led to a < 4-fold reduction in the expression of genes related to different stages of the tumorigenic process in HT29 cells, it should be noted that AURKA and E2F4 are involved in cell cycle regulation and transcription. The enzyme encoded by AURKA, aurora kinase A, is involved in cell cycle progression, and its function is vital to cell health^22^. E2FA codes for a transcription factor that acts as a repressor of regulatory elements for miRNAs^23^.

The present results also show that tyramine affects the expression of genes involved in the response to DNA damage, suggesting that it might be genotoxic towards intestinal cells. Tyramine induced the strong expression (>4-fold change) of four genes belonging to the growth-arrest and DNA damage (GADD) family genes: GADD45A (which codes for the growth-arrest and DNA damage-inducible alpha protein), GADD45G (which codes for the growth-arrest and DNA damage-inducible gamma protein), PPP1R15A (also known as GADD34, which codes for phosphatase 1 regulatory subunit 15A) and DDIT3 (also known as GADD153 or CHOP, which codes for the DNA damage-inducible transcript 3 protein)^24^. The expression of these genes is often increased in response to stressful growth-arrest conditions and during genotoxic stress (e.g., when exposed to DNA-damaging agents) and may lead to the arrest of the cell cycle, DNA repair, senescence, and, ultimately apoptosis^25,26^. All code for nuclear proteins that protect cells and ensure their survival^24^. Upon the detection of DNA damage, cells induce the expression of genes including the GADD family members, which arrest cell cycle progression at the G1/S or G2/M checkpoints, thus allowing for DNA repair before entry into the S and M phases. Moreover, the present results show that tyramine induces the significant overexpression of these genes in intestinal epithelial cells at concentrations easily reached in BA-rich foods such as cheese. Indeed, DDIT3 was significantly overexpressed at 1.82 mM tyramine (equivalent to 250 mg/kg), while GADD45G and PPP1R15A were significantly overexpressed at 3.65 mM tyramine (equivalent to 500 mg/kg). Further, GADD45A showed significant upregulation at 7.29 mM tyramine - equivalent to 1000 mg/kg, a concentration also reached in some cheeses^9,10,27^. Thus, a tyramine concentration as low as 250 mg/kg - which is easily reached in some fermented foods - might induce DNA damage in intestinal cells.

The BBC3 gene also showed significant upregulation in response to tyramine exposure. BBC3, also known as PUMA (a p53-upregulated modulator of apoptosis), codes for Bcl-2-binding component 3, an essential mediator of both p53-dependent and p53-independent apoptosis^28^. BBC3 is normally expressed at low levels, but is rapidly induced by genotoxic stress and toxins. In response to DNA damage, BBC3 is upregulated by the tumour suppressor p53^28^. However, BBC3 expression can also be increased by p53-independent stimuli. For instance, the treatment of primary murine thymocytes with dexamethasone, or subjecting HT29 cells to serum deprivation, induces the expression of BBC3 by a p53-independent cell death pathway^29^. The expression of TP53 (which codes for p53) was unaffected by tyramine exposure (see Supplementary Table 2S). This BA thus provides a cell death stimulus that operates via increasing the expression of BBC3 in a p53-independent manner. Using a HT29 cell culture model, our group has shown tyramine to induce necrosis rather than apoptosis^16^. However, apoptotic DNA fragmentation was seen in some 12% of cultures treated with 5.8 mM tyramine^16^. In the present work, BBC3 expression was significantly increased after treatment with 7.29 mM tyramine concentrations (about 1000 mg/kg). This could indicate that tyramine concentrations of 1000 mg/kg and above might induce both necrosis and apoptosis via the induction of BBC3 in a p53-independent manner.

High tyramine concentrations (2000 mg/kg) significantly repressed, although not dramatically (expression fold-change of <4), the transcription of some DNA-repair genes involved in MMR (i.e., MLH3 and PMS1; Supplementary Table S2) and NER (i.e., XPA and ERCC2 [Supplementary Table S2]; and ERCC3 [Supplementary Table S1]). The MMR system has an important role in maintaining genomic stability by correcting chromosomal errors that arise during DNA replication, recombination, and chemical damage^30^. Deficient MMR has been associated with an increased risk of developing colorectal^31^ and ovarian cancer^32^. MLH3 and PMS1 are counted among the mammalian MMR genes, and participate in repairing base-pair abnormalities^33^. In papillary thyroid cancer, oxidative stress causes heavy DNA damage and the downregulation of MMR-associated genes (including MLH3 and PMS1). The deficient MMR that ensues could play a part in the appearance of this malignancy^33^. Further, the NER system is the main pathway involved in the repair of damage that destabilizes the DNA helix, including the bulky DNA lesions formed by UV light and mutagenic compounds^34^. A loss of NER capacity has been associated with an increased risk of developing skin, breast and testicular cancer^35^. The downregulation of the NER-associated genes has been confirmed in some tumours. For instance, 20 canonical NER genes (including XPA, a central component of the NER complex, and ERCC2 [also known as XPD]) are downregulated in breast tumours^35^. XPA is also repressed in bladder cancer, in which it appears to increase the incidence of chromosome aberrations and promote carcinogenesis^36^. The reduced expression of ERCC3 in lymphocytes is associated with an increased risk of head and neck squamous cell carcinoma^37^. The present results therefore suggest that the high tyramine concentrations (2000 mg/kg) found in BA-rich food might promote carcinogenesis in intestinal epithelial cells by reducing the expression of some of the genes involved in the MMR and NER systems. The expression fold-reduction observed for MLH3, PMS1, XPA, ERCC2 and ERCC3 after exposure to high tyramine concentrations was quite low (between 2.19 and 3.94). It cannot be ruled out, however, that these relatively small changes are without biological significance.

The present results also show that tyramine has a greater effect on HT29 cells than either histamine or putrescine; only the expression of four genes (ARNT, CFLAR, CPT2, and E2F4) was significantly modified by histamine, and only two (CPT2 and PPP1R15A) by putrescine, and all by less than 4-fold. These results underscore the health risk associated with the consumption of tyramine, which has already been reported the most cytotoxic of the BA commonly present in food^16^ (del Rio *et al*., submitted).

The highest concentration of tyramine used in this study correlated with the highest accumulations of these BA ever reported in food^10^ and therefore, it would mimic the greatest risk of intoxication. The real concentrations of ingested tyramine that reach the intestine would be lower due to a dilution effect during food digestion and to its detoxification in the intestinal mucosa by monoamine oxidases^1^. However, in food safety the precautionary principle must be applied and therefore, the worst possible scenarios have to be contemplated. For example, it is well known that certain foods have high concentrations of more than one BA, which are the substrate of the same monoamine oxidase and can saturate the capacity of the detoxifying system^1,2^. Therefore, it cannot be ruled out that the concentrations of tyramine found to be genotoxic for intestinal cell cultures, could reach the intestinal mucosa and may constitute a hazard for human health. Moreover, people with dysfunctional BA-degrading systems or taking monoamine oxidase inhibitor drugs might be even at greater risk. It should be also taken into account that the observed toxic effects of ingested tyramine could be reduced due to the so call “matrix effect” of food, but they could also be enhanced due to the presence of other components such as alcoholic beverages, which potentiates the toxic effect of BA^2^.

In conclusion, the present results suggest that tyramine, at concentrations easily found in BA-rich food, might be genotoxic for intestinal cells in culture, causing DNA damage that results in cell cycle arrest. The apparent deficiency caused by tyramine in the MMR and NER DNA repair mechanisms, combined with possible abnormal lipid accumulation due the repression of fatty acid ß-oxidation, might jeopardize the stability of the genome and the metabolic functionality of exposed intestinal cells. Ultimately, exposure could lead to cell death by both apoptotic and necrotic mechanisms. Further, tyramine might play a part in intestinal carcinogenesis; this needs to be further studied. These present findings raise further concerns regarding the concentration of tyramine that can be permitted in foods.

## Methods

### Cell line cultures

The human intestinal cell line HT29 (ECACC 91072201) - purchased from the European Collection of Cell Cultures (Salisbury, UK) - was used to test the effect of tyramine, histamine and putrescine on the expression of cancer-related and DNA damage signalling-related genes. 10^5^ cells were seeded in 12-well plates (BD Falcon, BD Biosciences, NJ, USA) using complete McCoy’s 5a medium (i.e., McCoy’s 5a medium supplemented with 10% heat-inactivated foetal bovine serum, 50 µg/ml penicillin, 50 µg/ml streptomycin, 50 µg/ml gentamicin, and 1.25 µg/ml amphotericin B). All media and reagents were purchased from Sigma-Aldrich (Madrid, Spain). Plates were incubated at 37 °C in a 5% CO_2_ atmosphere within a CO_2_-Series SL Waterjacked CO_2_ Incubator (Sheldon Manufacturing Inc., Conrnelius, OR, USA) until cultures reached the confluent (monolayer) state (12 ± 1 days post-seeding; about 10^7^ cells/ml). Stock solutions of tyramine (tyramine hydrochloride) (Acros Organics, Thermo Fisher Scientific, Madrid, Spain), histamine (histamine hydrochloride) and putrescine (putrescine dihydrochloride) (all from Sigma-Aldrich), were dissolved in water adjusted to pH 7 and filter-sterilized using Acrodisc Syringe Filters with a HT Tuffryn (polysulphone) membrane (Pall Corporation, Ann Arbor, MI, USA). Confluent cells were treated with 1 ml complete McCoy’s 5a medium supplemented with one of five concentrations of tyramine (0.91, 1.82, 3.65, 7.29 and 14.58 mM), 17.99 mM of histamine, or 17.02 mM putrescine for 6 h. Control cells were treated with 1 ml complete McCoy’s 5a medium. The culture medium was then removed and 500 μl of RNAprotect Cell Reagent (Qiagen, Hilden, Germany) added to stabilize the RNA. Cells were then collected and stored at −80 °C.

### RNA extraction and cDNA synthesis

Total RNA was extracted from replicate samples using the RNeasy Mini Kit (Qiagen) following the manufacturer’s recommendations. Total RNA from each replicate was eluted in 50 µL of nuclease-free water. RNA samples were treated with DNase using the RNase-Free DNase Set (Qiagen), following the manufacturers recommendations, to eliminate any DNA contamination. The DNase-treated RNA was further cleaned according to the RNA Cleanup protocol described in the RNeasy Mini Kit manual. The quality of RNA was determined in 1% agarose gels containing 1% sodium hypochlorite. The concentration of extracted RNA was determined using an Epoch Microplate spectrophotometer (BioTek Instruments, Inc., Winoskii, VT, USA). cDNA was synthesised using 1 µg of total RNA and the RT^2^ First Strand Kit (Qiagen) following the manufacturer’s recommendations.

### RT^2^ Profiler™ PCR Arrays

cDNA samples were analyzed for the expression of 84 genes representative of biological pathways involved in cell transformation and tumorigenesis in humans, and for 84 genes involved in DNA damage-signalling pathways, using two RT^2^ Profiler™ PCR Arrays (Qiagen): the Human Cancer Pathway Finder (PAHS-033ZC) and Human DNA Damage Signaling Pathway (PAHS-029ZC) Arrays respectively. cDNA template was combined with RT^2^ SYBR Green qPCR Master Mix (Qiagen) and water. Equal aliquots of this mixture (25 µl) were added to each well of the PCR array plate loaded with the predispensed gene-specific primer sets. Amplification and detection were performed using an ABI Prism Fast 7500 sequence detection system (Applied Biosystems, Carlsbad, CA, USA). PCR array quantification was based on the threshold cycle (C_T_) number. The baseline was defined by choosing the automated baseline option of the cycler; the threshold values were the same across all RT^2^ Profiler PCR Array runs in the same analysis. A gene was considered undetectable at C_T_ > 35, according to the array user manuals. C_T_ values were exported to a blank Excel spreadsheet. Web-based PCR array data analysis software (www.SABiosciences.com/pcrarraydataanalysis.php) was used for ∆∆C_T_–based fold-change calculations using real-time data collected in triplicate (two biological and one technical replicate). Samples were manually normalized using the geometric mean for the GAPDH and RPLP0 (housekeeping genes). A gene was considered differentially expressed when significance was <0.05. Volcano plots, which displays statistical significance vs. fold-change on the y- and x-axes respectively, were used to identify significant gene expression changes.

### Real-time qPCR

qPCR was performed to check the absence of genomic DNA in the RNA samples and to confirm the PCR array results. Samples were analyzed using an ABI Prism Fast 7500 sequencer. Reactions were performed in 25 µl reaction volumes containing Power SYBR green PCR Master Mix (Applied Biosystems) and primers at a final concentration of 0.2 µM. Cycling was performed under the device’s default settings. To check the quality of the RNA samples, the primer pair ACTB-Fw and ACTB-Rw was used (Table 1). RNA samples were considered free of genomic DNA when a C_T_ value of >30 was obtained.

**Table 1:** Primers used for the quantification of gene expression by reverse transcription quantitative PCR (RT-qPCR).

Gene	Primer	RefSeq Accession no.*	Source
ACTB^a^	ACTB-Fw: 5′ TTGTTACAGGAAGTCCCTTGCC 3′		^39^
	ACTB-Rw: 5′ATGCTATCACCTCCCCTGTGTG 3′		^39^
BBC3^a^	RT² qPCR Primer Assay for Human BBC3	NM_014417.4	Qiagen
CPT2^a^	RT² qPCR Primer Assay for Human CPT2	NM_000098.2	Qiagen
DDIT3^a^	RT² qPCR Primer Assay for Human DDIT3	NM_001195053	Qiagen
GADD45A^a^	RT² qPCR Primer Assay for Human GADD45A	NM_001924.3	Qiagen
GADD45G^a^	RT² qPCR Primer Assay for Human GADD45G	NM_006705.3	Qiagen
PPP1R15A^a^	RT² qPCR Primer Assay for Human PPP1R15A	NM_014330.3	Qiagen
GAPDH^b^	RT² qPCR Primer Assay for Human GAPDH	NM_002046.5	Qiagen
RPLP0^b^	RT² qPCR Primer Assay for Human RPLP0	NM_001002.3	Qiagen

^a^Target genes.

^b^Reference genes.

*The RefSeq accession no. refers to the sequence used to design the RT^2^ qPCR Primer Assay.

cDNA samples obtained from BA-treated HT29 cells were analyzed using predesigned primers (Qiagen; see Table 1) at final concentrations of 0.2 µM: PPH02204C for BBC3, PPH15572A for CPT2, PPH00310A for DDIT3, PPH00148B for GADD45A, PPH02207A for GADD45G, and PPH02081E for PPP1R15A; the housekeeping gene primers used were PPH00150F for GAPDH and PPH21138F for RPLP0. Relative gene expression was calculated using the ΔΔCt comparative method^38^. For each condition, qPCR analysis was performed on RNA samples from four different cultures (two biological replicates and two technical replicates).

### Statistical analysis

The analysis of the gene expression data returned by the RT^2^ Profiler PCR Arrays was performed using RT^2^ Profiler PCR Array Data Analysis v.3.5 software (Qiagen). Data were calculated using values for one technical and two biological replicates. For qPCR analysis, means ± standard deviations were calculated from two biological replicates (each in duplicate). The Student *t*-test was used to examine differences between groups. Significance was set at *p* < 0.05.

## Electronic supplementary material

Table 1S and Table 2S
