# Supplementary material for: An altered gene expression profile in tyramine-exposed intestinal cell cultures supports the genotoxicity of this biogenic amine at dietary concentrations
Source: Sci Rep. 2018 Nov 19;8:17038. doi: 10.1038/s41598-018-35125-9 (PMC6242974; doi:10.1038/s41598-018-35125-9)
Supplement: Supplementary file 1 — Table 1S and Table 2S [file 41598_2018_35125_MOESM1_ESM.pdf]

Supplementary information

An altered gene expression profile in tyramine-exposed intestinal cell cultures supports the genotoxicity of this biogenic amine at dietary concentrations

Beatriz del Rio, Begoña Redruello, Victor Ladero, Santiago Cal, Alvaro Obaya and Miguel A. Alvarez

Table 1S. Gene expression analysis of tyramine-, histamine- and putrescine-exposed HT29 cells compared to untreated HT29 cells using the RT^2 Profiler™ PCR Arrays Human Cancer Pathway Finder array (PAHS-033ZC, Qiagen).

Gene symbol	Description	Tma/Control		Hma/Control		Put/Control	
		Fold change	*p*-value	Fold change	*p*-value	Fold change	*p*-value
**Angiogenesis**							
ANGPT1	Angiopoietin 1	-1.88	0.1243	-1.49	0.3312	-1.930	0.0922
ANGPT2	Angiopoietin 2	-1.58	0.2391	2.91	0.0536	2.160	0.1380
CCL2	Chemokine (C-C motif) ligand 2	1.37	0.2061	1.14	0.5710	-1.040	0.7618
FGF2	Fibroblast growth factor 2 (basic)	-1.64	0.0249	1.13	0.5383	-1.390	0.4600
FLT1	Fms-related tyrosine kinase 1	1.37	0.2061	-1.08	0.8743	-1.040	0.7618
KDR	Kinase insert domain receptor (a type III receptor tyrosine kinase)	1.37	0.2061	-1.08	0.8743	-1.040	0.7618
PGF	Placental growth factor	4.87	0.0672	1.96	0.1298	-1.210	0.5668
SERPINF1	Serpin peptidase inhibitor, clade F, member 1	-2.22	0.0698	1.25	0.4446	1.420	0.2546
TEK	TEK tyrosine kinase, endothelial	-4.90	0.0574	-2.12	0.2231	-1.560	0.2521
VEGFC	Vascular endothelial growth factor C	1.37	0.2061	-1.08	0.8743	-1.040	0.7618
**Apoptosis**							
APAF1	Apoptotic peptidase activating factor 1	1.41	0.5716	1.30	0.6237	1.390	0.5301
BCL2L11	BCL2-like 11 (apoptosis facilitator)	2.70	0.1541	1.19	0.5451	1.040	0.9924
BIRC3	Baculoviral IAP repeat containing 3	-2.31	0.1213	-1.22	0.7010	-1.150	0.9962
CASP2	Caspase 2, apoptosis-related cysteine peptidase	-1.27	0.5067	-1.08	0.8029	-1.310	0.5039
CASP7	Caspase 7, apoptosis-related cysteine peptidase	-2.62	0.0356	-1.31	0.2277	-1.060	0.8923

CASP9	Caspase 9, apoptosis-related cysteine peptidase	-1.57	0.2327	-1.04	0.8403	-1.220	0.2304
CFLAR	CASP8 and FADD-like apoptosis regulator	1.04	0.7391	-1.40	0.0469	-1.460	0.0635
FASLG	Fas ligand (TNF superfamily, member 6)	1.37	0.2061	-1.08	0.8743	-1.040	0.7618
NOL3	Nucleolar protein 3 (apoptosis repressor with CARD domain)	-2.27	0.1475	-1.18	0.6041	-1.250	0.3566
XIAP	X-linked inhibitor of apoptosis	-1.79	0.0798	1.00	0.9677	1.070	0.5874
**Cell Cycle**							
AURKA	Aurora kinase A	-2.57	0.0493	-1.26	0.3667	-1.320	0.4206
CCND2	Cyclin D2	1.39	0.1694	-1.08	0.8743	-1.040	0.7618
CCND3	Cyclin D3	1.12	0.8521	-1.28	0.5541	-1.320	0.4865
CDC20	Cell division cycle 20 homologue (*S. cerevisiae*)	-1.50	0.0750	-1.17	0.3774	-1.200	0.6298
E2F4	E2F transcription factor 4, p107/p130-binding	-1.46	0.0274	-1.36	0.0250	-1.320	0.0698
MCM2	Minichromosome maintenance complex component 2	-3.29	0.1065	-2.18	0.2231	-1.730	0.4511
MKI67	Antigen identified by monoclonal antibody Ki-67	-1.51	0.3500	-1.05	0.7903	-1.340	0.5063
SKP2	S-phase kinase-associated protein 2 (p45)	-4.04	0.0599	-1.67	0.2235	-1.560	0.2710
STMN1	Stathmin 1	1.26	0.4939	-1.03	0.9435	-1.180	0.7388
WEE1	WEE1 homologue (*S. pombe*)	1.22	0.5274	-1.45	0.4050	-1.320	0.5944
**Cellular Senescence**							
BMI1	BMI1 polycomb ring finger oncogene	-7.68	0.4092	1.11	0.4282	-1.050	0.5428
ETS2	V-Ets erythroblastosis virus E26 oncogene homologue 2 (avian)	-3.15	0.0291	-1.11	0.6217	-1.080	0.6165
IGFBP3	Insulin-like growth factor binding protein 3	1.37	0.2061	-1.08	0.8743	-1.040	0.7618
IGFBP5	Insulin-like growth factor binding protein 5	1.37	0.2061	-1.08	0.8743	-1.040	0.7618
IGFBP7	Insulin-like growth factor binding protein 7	1.05	0.9042	1.18	0.7192	1.470	0.3433
MAP2K1	Mitogen-activated protein kinase 1	-1.21	0.3543	1.02	0.9950	-1.050	0.7217
MAP2K3	Mitogen-activated protein kinase 3	1.03	0.9574	-1.10	0.8726	-1.410	0.4034
MAPK14	Mitogen-activated protein kinase 14	1.01	0.8941	-1.07	0.6595	1.030	0.8589
SERPINB2	Serpin peptidase inhibitor, clade B (ovalbumin), member 2	1.37	0.2061	-1.08	0.8743	-1.040	0.7618
SOD1	Superoxide dismutase 1, soluble	1.13	0.1821	-1.07	0.7523	-1.120	0.4288
TBX2	T-box 2	1.27	0.3702	-1.16	0.6779	-1.120	0.5295
**DNA Damage & Repair**							
DDB2	Damage-specific DNA binding protein 2, 48kDa	1.18	0.7198	1.09	0.9518	-1.040	0.8413
**DDIT3**	DNA-damage-inducible transcript 3	22.37	0.0924	3.52	0.1649	1.000	0.9946
ERCC3	Excision repair cross-complementing rodent repair deficiency, complementation group 3	-2.80	0.0127	-1.09	0.2256	-1.060	0.7288
ERCC5	Excision repair cross-complementing rodent repair deficiency, complementation group 5	-1.64	0.5008	1.01	0.9620	-1.140	0.4275
**GADD45G**	Growth arrest and DNA-damage-inducible, gamma	6.63	0.0122	1.53	0.0693	1.260	0.2258
LIG4	Ligase IV, DNA, ATP-dependent	1.02	0.8601	1.36	0.1682	1.060	0.8295
POLB	Polymerase (DNA directed), beta	1.58	0.0228	-1.04	0.7872	-1.060	0.6436
**PPP1R15A**	Protein phosphatase 1, regulatory (inhibitor) subunit 15A	7.98	0.0035	1.32	0.3660	-1.600	0.0221
**Epithelial-to-Mesenchymal Transition (EMT)**							
CDH2	Cadherin 2, type 1, N-cadherin (neuronal)	1.37	0.2061	-1.08	0.8743	-1.040	0.7618
DSP	Desmoplakin	-1.15	0.6654	-1.21	0.3742	-1.200	0.4693

FOXC2	Forkhead box C2 (MFH-1, mesenchyme forkhead 1)	4.25	0.0971	1.19	0.5013	1.120	0.6930
GSC	Goosecoid homeobox	1.37	0.2061	-1.08	0.8743	-1.040	0.7618
KRT14	Keratin 14	1.20	0.1650	-1.39	0.2777	-1.170	0.3314
OCLN	Occludin	1.90	0.0068	1.15	0.5294	1.070	0.7531
SNAI1	Snail homologue 1 (Drosophila)	7.72	0.1157	1.07	0.7528	1.100	0.6270
SNAI2	Snail homologue 2 (Drosophila)	1.37	0.2061	-1.08	0.8743	-1.040	0.7618
SNAI3	Snail homologue 3 (Drosophila)	1.19	0.6680	1.45	0.3774	1.700	0.1641
SOX10	SRY (sex determining region Y)-box 10	1.93	0.1528	-1.04	0.9625	1.240	0.4950
**Hypoxia signalling**							
ADM	Adrenomedullin	3.19	0.0989	1.10	0.6212	1.060	0.7108
ARNT	Aryl hydrocarbon receptor nuclear translocator	1.06	0.6516	1.81	0.0167	1.490	0.1144
CA9	Carbonic anhydrase IX	-1.45	0.0806	1.03	0.7780	-1.110	0.6548
EPO	Erythropoietin	1.37	0.2061	-1.07	0.8980	1.740	0.1819
HMOX1	Heme oxygenase (decycling) 1	13.53	0.0519	1.17	0.8034	1.180	0.7442
LDHA	Lactate dehydrogenase A	-2.08	0.0908	1.07	0.9893	1.060	0.9719
SLC2A1	Solute carrier family 2, member 1	-2.24	0.1029	-1.68	0.4578	-1.350	0.6493
**Metabolism**							
ACLY	ATP citrate lyase	1.37	0.2061	-1.08	0.8743	-1.040	0.7618
ACSL4	Acyl-CoA synthetase long-chain family member 4	-1.50	0.1674	1.10	0.6949	-1.050	0.7660
ATP5A1	ATP synthase, H^+ transporting, mitochondrial F1 complex, alpha subunit 1, cardiac muscle	-1.87	0.0130	-1.15	0.4979	-1.090	0.5969
COX5A	Cytochrome c oxidase subunit Va	-1.18	0.1617	-1.11	0.5265	-1.120	0.4827
**CPT2**	Carnitine palmitoyltransferase 2	-9.09	0.0002	-2.95	0.0017	-2.180	0.0036
G6PD	Glucose-6-phosphate dehydrogenase	-1.24	0.9599	1.38	0.5701	1.310	0.6428
GPD2	Glycerol-3-phosphate dehydrogenase 2 (mitochondrial)	-2.25	0.0553	-1.62	0.1418	-1.500	0.2296
LPL	Lipoprotein lipase	1.37	0.2061	-1.08	0.8743	-1.040	0.7618
PFKL	Phosphofructokinase, liver	-1.24	0.3087	-1.61	0.1607	-1.480	0.1378
UQCRFS1	Ubiquinol-cytochrome c reductase, Rieske iron-sulphur polypeptide 1	-1.35	0.0502	-1.09	0.4166	-1.080	0.6013
**Telomeres & Telomerase**							
DKC1	Dyskeratosis congenita 1, dyskerin	-1.70	0.2377	-1.17	0.5911	-1.270	0.4944
PINX1	PIN2/TERF1 interacting, telomerase inhibitor 1	-2.31	0.3755	1.04	0.8849	-1.150	0.6328
TEP1	Telomerase-associated protein 1	-1.34	0.3781	-1.35	0.2554	-1.190	0.4046
TERF1	Telomeric repeat binding factor (NIMA-interacting) 1	-1.52	0.4448	-1.21	0.4676	-1.140	0.5530
TERF2IP	Telomeric repeat binding factor 2, interacting protein	2.84	0.1859	-1.06	0.8169	-1.270	0.4599
TINF2	TERF1 (TRF1)-interacting nuclear factor 2	-1.80	0.0690	-1.40	0.0502	-1.150	0.1582
TNKS	Tankyrase, TRF1-interacting ankyrin-related ADP-ribose polymerase	-1.31	0.3000	-1.40	0.2539	-1.520	0.2922
TNKS2	Tankyrase, TRF1-interacting ankyrin-related ADP-ribose polymerase 2	-2.08	0.1044	-1.14	0.5372	-1.020	0.9441

Genes were sorted by Functional Gene Grouping as outlined by the RT² Profiler™ PCR Array Human Cancer PathwayFinder™ Kit. Gene expression levels highlighted in boldface were confirmed by real-time qPCR analysis. Tma: tyramine; Hma: histamine; Put: putrescine.

Table 2S. Gene expression of tyramine-exposed HT29 cells compared to untreated HT29 cells using the RT^2 PCR Human DNA Damage Signaling Pathway array (PAHS-029ZC, Qiagen).

Gene symbol	Description	Tma/Control	
		Fold change	*p*-value
**ATM / ATR signalling**			
ATM	Ataxia telangiectasia mutated	-1.83	0.2063
ATR	Ataxia telangiectasia and Rad3 related	-2.26	0.0531
ATRIP	ATR interacting protein	-2.73	0.0454
BARD1	BRCA1 associated RING domain 1	-3.20	0.1430
BRCA1	Breast cancer 1, early onset	-1.92	0.2411
CDC25A	Cell division cycle 25 homologue A (*S. pombe*)	-1.47	0.7119
CHEK1	CHK1 checkpoint homologue (*S. pombe*)	-1.38	0.4933
CHEK2	CHK2 checkpoint homologue (*S. pombe*)	-1.14	0.6314
CSNK2A2	Casein kinase 2, alpha prime polypeptide	1.47	0.1013
FANCD2	Fanconi anaemia, complementation group D2	-1.33	0.4814
H2AFX	H2A histone family, member X	1.03	0.8531
HUS1	HUS1 checkpoint homologue (*S. pombe*)	-1.52	0.1916
MDC1	Mediator of DNA-damage checkpoint 1	-1.56	0.2740
PARP1	Poly (ADP-ribose) polymerase 1	-2.22	0.1493
RAD1	RAD1 homologue (*S. pombe*)	1.07	0.8671
RAD17	RAD17 homologue (*S. pombe*)	-1.83	0.0860
RAD50	RAD50 homologue (*S. cerevisiae*)	-2.12	0.1005
RAD9A	RAD9 homologue A (*S. pombe*)	1.10	0.8714
RBBP8	Retinoblastoma binding protein 8	-1.25	0.5131
RNF168	Ring finger protein 168	1.88	0.0783
RNF8	Ring finger protein 8	1.23	0.3715
SMC1A	Structural maintenance of chromosomes 1A	-1.07	0.6662
TOPBP1	Topoisomerase (DNA) II binding protein 1	-1.53	0.2681
TP53	Tumour protein p53	-2.38	0.1091
**DNA Damage & Repair**			
CDK7	Cyclin-dependent kinase 7	1.65	0.0120
DDB1	Damage-specific DNA binding protein 1, 127kDa	-1.33	0.1018
DDB2	Damage-specific DNA binding protein 2, 48kDa	1.18	0.8008
ERCC1	Excision repair cross-complementing rodent repair deficiency, complementation group 1	-1.34	0.0649
ERCC2	Excision repair cross-complementing rodent repair deficiency, complementation group 2	-2.44	0.0259
LIG1	Ligase I, DNA, ATP-dependent	-1.89	0.2830
NTHL1	Nth endonuclease III-like 1 (*E. coli*)	-1.50	0.1470
OGG1	8-oxoguanine DNA glycosylase	-1.03	0.8552

PCNA	Proliferating cell nuclear antigen	-1.69	0.2164
PNKP	Polynucleotide kinase 3'-phosphatase	-1.52	0.0998
RPA1	Replication protein A1, 70kDa	-2.17	0.0614
SIRT1	Sirtuin 1	4.42	0.0645
TP53	Tumour protein p53	-2.38	0.1091
XPA	Xeroderma pigmentosum, complementation group A	-2.19	0.0136
XPC	Xeroderma pigmentosum, complementation group C	-2.17	0.1902
**Base Excision Repair (BER)**			
APEX1	APEX nuclease (multifunctional DNA repair enzyme) 1	-1.22	0.1716
FEN1	Flap structure-specific endonuclease 1	-1.72	0.1985
LIG1	Ligase I, DNA, ATP-dependent	-1.89	0.2830
MBD4	Methyl-CpG binding domain protein 4	1.11	0.7889
MPG	N-methylpurine-DNA glycosylase	-1.38	0.0541
NTHL1	Nth endonuclease III-like 1 (*E. coli*)	-1.50	0.1470
OGG1	8-oxoguanine DNA glycosylase	-1.03	0.8552
PARP1	Poly (ADP-ribose) polymerase 1	-2.22	0.1493
PCNA	Proliferating cell nuclear antigen	-1.69	0.2164
TP53	Tumour protein p53	-2.38	0.1091
UNG	Uracil-DNA glycosylase	-1.69	0.1833
XRCC1	X-ray repair complementing defective repair in Chinese hamster cells 1	-1.45	0.2931
**Mismatch Repair (NMR)**			
ABL1	C-abl oncogene 1, non-receptor tyrosine kinase	1.04	0.7511
EXO1	Exonuclease 1	-1.37	0.3610
MLH1	MutL homologue 1, colon cancer, nonpolyposis type 2 (*E. coli*)	-1.97	0.0770
MLH3	MutL homologue 3 (*E. coli*)	-2.57	0.0115
MSH2	MutS homologue 2, colon cancer, nonpolyposis type 1 (*E. coli*)	-2.49	0.1088
MSH3	MutS homologue 3 (*E. coli*)	-1.15	0.5562
PCNA	Proliferating cell nuclear antigen	-1.69	0.2164
PMS1	PMS1 postmeiotic segregation increased 1 (*S. cerevisiae*)	-3.95	0.0460
PMS2	PMS2 postmeiotic segregation increased 2 (*S. cerevisiae*)	-1.44	0.0948
TP73	Tumour protein p73	-1.84	0.2684
**Double-Strand Break (DSB) Repair**			
ATM	Ataxia telangiectasia mutated	-1.83	0.2063
BLM	Bloom syndrome, RecQ helicase-like	-3.59	0.1165
BRCA1	Breast cancer 1, early onset	-1.92	0.2411
CHEK1	CHK1 checkpoint homologue (*S. pombe*)	-1.38	0.4933
H2AFX	H2A histone family, member X	1.03	0.8531
HUS1	HUS1 checkpoint homologue (*S. pombe*)	-1.52	0.1916
LIG1	Ligase I, DNA, ATP-dependent	-1.89	0.2830
MDC1	Mediator of DNA-damage checkpoint 1	-1.56	0.2740
MLH1	MutL homologue 1, colon cancer, nonpolyposis type 2 (*E. coli)*	-1.97	0.0770
MRE11A	MRE11 meiotic recombination 11 homologue A (*S. cerevisiae*)	-1.34	0.4849
NBN	Nibrin	-1.22	0.2421

PRKDC	Protein kinase, DNA-activated, catalytic polypeptide	-1.66	0.1150
RAD50	RAD50 homologue (*S. cerevisiae*)	-2.12	0.1005
RAD51	RAD51 homologue (*S. cerevisiae*)	-1.44	0.3643
RPA1	Replication protein A1, 70kDa	-2.17	0.0614
TP53BP1	Tumour protein p53 binding protein 1	-1.05	0.7563
XRCC2	X-ray repair complementing defective repair in Chinese hamster cells 2	-3.82	0.1453
XRCC6	X-ray repair complementing defective repair in Chinese hamster cells 6	-1.30	0.2698
**Other DNA Repair Genes**			
ATR	Ataxia telangiectasia and Rad3 related	-2.26	0.0531
ATRIP	ATR interacting protein	-2.73	0.0454
ATRX	Alpha thalassaemia/mental retardation syndrome X-linked	-1.52	0.2341
BARD1	BRCA1 associated RING domain 1	-3.20	0.1430
BRIP1	BRCA1 interacting protein C-terminal helicase 1	-2.12	0.2582
CHEK2	CHK2 checkpoint homologue (*S. pombe*)	-1.14	0.6314
CIB1	Calcium and integrin binding 1 (calmyrin)	-1.15	0.4901
CRY1	Cryptochrome 1 (photolyase-like)	2.14	0.0496
FANCA	Fanconi anaemia, complementation group A	-1.09	0.7782
FANCD2	Fanconi anaemia, complementation group D2	-1.33	0.4814
FANCG	Fanconi anaemia, complementation group G	1.20	0.9450
**GADD45A**	Growth arrest and DNA-damage-inducible, alpha	4.94	0.0020
**GADD45G**	Growth arrest and DNA-damage-inducible, gamma	6.64	0.0131
RAD1	RAD1 homologue (*S. pombe*)	1.07	0.8671
RAD17	RAD17 homologue (*S. pombe*)	-1.83	0.0860
RAD18	RAD18 homologue (*S. cerevisiae*)	-2.03	0.0659
RAD21	RAD21 homologue (*S. pombe*)	-1.28	0.4098
RAD51B	RAD51 homologue B (*S. cerevisiae*)	1.14	0.7212
RAD9A	RAD9 homologue A (*S. pombe*)	1.10	0.8714
RBBP8	Retinoblastoma binding protein 8	-1.25	0.5131
REV1	REV1 homologue (*S. cerevisiae*)	-1.10	0.9557
RNF168	Ring finger protein 168	1.88	0.0783
RNF8	Ring finger protein 8	1.23	0.3715
SMC1A	Structural maintenance of chromosomes 1A	-1.07	0.6662
SUMO1	SMT3 suppressor of Mif Two 3 homologue 1 (*S. cerevisiae*)	1.00	0.9842
TOPBP1	Topoisomerase (DNA) II binding protein 1	-1.53	0.2681
XRCC3	X-ray repair complementing defective repair in Chinese hamster cells 3	-1.76	0.3274
**Apoptosis**			
ATM	Ataxia telangiectasia mutated	-1.83	0.2063
ABL1	C-abl oncogene 1, non-receptor tyrosine kinase	1.04	0.7511
ATM	Ataxia telangiectasia mutated	-1.83	0.2063
BARD1	BRCA1 associated RING domain 1	-3.20	0.1430
BAX	BCL2-associated X protein	-1.28	0.2157
**BBC3**	BCL2 binding component 3	22.23	0.0055
BRCA1	Breast cancer 1, early onset	-1.92	0.2411
CDKN1A	Cyclin-dependent kinase inhibitor 1A (p21, Cip1)	7.59	0.1030

CHEK2	CHK2 checkpoint homologue (*S. pombe*)	-1.14	0.6314
CIB1	Calcium and integrin binding 1 (calmyrin)	-1.15	0.4901
CSNK2A2	Casein kinase 2, alpha prime polypeptide	1.47	0.1013
**PPP1R15A**	Protein phosphatase 1, regulatory (inhibitor) subunit 15A	10.19	0.0017
PRKDC	Protein kinase, DNA-activated, catalytic polypeptide	-1.66	0.1150
RAD21	RAD21 homologue (*S. pombe*)	-1.28	0.4098
RAD9A	RAD9 homologue A (*S. pombe*)	1.10	0.8714
SIRT1	Sirtuin 1	4.42	0.0645
TP53	Tumour protein p53	-2.38	0.1091
TP73	Tumour protein p73	-1.84	0.2684
**Cell Cycle**			
ATM	Ataxia telangiectasia mutated	-1.83	0.2063
ATR	Ataxia telangiectasia and Rad3 related	-2.26	0.0531
ATRIP	ATR interacting protein	-2.73	0.0454
CDC25A	Cell division cycle 25 homologue A (*S. pombe*)	-1.47	0.7119
CDC25C	Cell division cycle 25 homologue C (*S. pombe*)	1.01	0.9110
CDK7	Cyclin-dependent kinase 7	1.65	0.0120
CDKN1A	Cyclin-dependent kinase inhibitor 1A (p21, Cip1)	7.59	0.1030
CHEK1	CHK1 checkpoint homologue (*S. pombe*)	-1.38	0.4933
CHEK2	CHK2 checkpoint homologue (*S. pombe*)	-1.14	0.6314
**DDIT3**	DNA-damage-inducible transcript 3	20.20	0.0927
MAPK12	Mitogen-activated protein kinase 12	-1.31	0.3922
MCPH1	Microcephalin 1	1.41	0.0537
MDC1	Mediator of DNA-damage checkpoint 1	-1.56	0.2740
PPM1D	Protein phosphatase, Mg2+/Mn2+ dependent, 1D	3.94	0.1147
**PPP1R15A**	Protein phosphatase 1, regulatory (inhibitor) subunit 15A	10.19	0.0017
TP53	Tumour protein p53	-2.38	0.1091
TP73	Tumour protein p73	-1.84	0.2684

Genes were sorted by Functional Gene Grouping as outlined by the RT² Profiler™ PCR Array Human Cancer PathwayFinder™ Kit. Gene expression levels highlighted in boldface were confirmed by real-time qPCR analysis. Tma: tyramine.
